# 2D MXenes Embedded Perovskite Hydrogels for Efficient and Stable Solar Evaporation

**DOI:** 10.1002/gch2.202300091

**Published:** 2023-07-20

**Authors:** Naila Arshad, Muhammad Sultan Irshad, M. Sohail Asghar, Muneerah Alomar, Junyang Tao, M. A. K. Yousaf Shah, Xianbao Wang, Jinming Guo, S. Wageh, Omar A. Al‐Hartomy, Abul Kalam, Yabin Hao, Zhengbiao Ouyang, Han Zhang

**Affiliations:** ^1^ Collaborative Innovation Centre for Optoelectronic Science & Technology International Collaborative Laboratory of 2D Materials for Optoelectronics Science and Technology of Ministry of Education Institute of Microscale Optoelectronics Shenzhen University Shenzhen 518060 P. R. China; ^2^ Interdisciplinary Center of High Magnetic Field Physics College of Physics and Optoelectronic Engineering Shenzhen University Shenzhen 518060 P. R. China; ^3^ School of Materials Science and Engineering Hubei University Wuhan 430062 P. R. China; ^4^ Department of Physics College of Sciences Princess Nourah bint Abdulrahman University P. O. Box 84428 Riyadh 11671 Saudi Arabia; ^5^ School of Energy and Environment Southeast University No. 2 Si Pai Lou Nanjing 210096 China; ^6^ Department of Physics Faculty of Science King Abdulaziz University Jeddah 21589 Saudi Arabia; ^7^ Research Center for Advanced Materials Science (RCAMS) King Khalid University P. O. Box 9004 Abha 61413 Saudi Arabia

**Keywords:** fresh water, LSCF, MXenes, perovskite, photothermal, solar energy, water scarcity

## Abstract

Solar evaporation is a facile and promising technology to efficiently utilize renewable energy for freshwater production and seawater desalination. Here, the fabrication of self‐regenerating hydrogel composed of 2D‐MXenes nanosheets embedded in perovskite La _0.6_Sr _0.4_Co _0.2_Fe _0.8_O_3−_ *_δ_* (LSCF)/polyvinyl alcohol hydrogels for efficient solar‐driven evaporation and seawater desalination is reported. The mixed dimensional LSCF/Ti_3_C_2_ composite features a localized surface plasmonic resonance effect in the polymeric network of polyvinyl alcohol endows excellent evaporation rates (1.98 kg m^−2^ h^−1^) under 1 k Wm^−2^ or one sun solar irradiation ascribed by hydrophilicity and broadband solar absorption (96%). Furthermore, the long‐term performance reveals smooth mass change (13.33 kg m^−2^) during 8 h under one sun. The composite hydrogel prompts the dilution of concentrated brines and redissolves it back to water (1.2 g NaCl/270 min) without impeding the evaporation rate without any salt‐accumulation. The present research offers a substantial opportunity for solar‐driven evaporation without any salt accumulation in real‐life applications.

## Graphical abstract

It is reported 2D MXenes embedded perovskite hydrogels which endow stable solar‐driven evaporation (1.98 kg m^−2^ h^−1^) along with 96 % solar absorption. Besides, the hydratable polymeric network achieves excellent salt‐resistant capability.

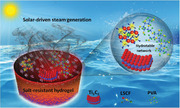

## 1 Introduction

Water scarcity has increased severalfold in the past few decades with the advancement in the lifestyle and industrial sectors. The commercial energy sources, i.e., fragmentation of exhaustive and polluting fossil fuels resulted in expediting global warming and declined the quality of human life.^[^ ^1^, ^2^, ^3^ ^]^ Additionally, the diminishing fossil fuel reserves have increased the demand for inexhaustive energy sources. Traditional water desalination technologies cost intensive energy consumption with the simultaneous inflation of global warming are not plausible for long‐term sustainability at the expense of exasperating energy issues and environmental degradation.^[^ ^4^, ^5^ ^]^ Therefore, scientists are encouraged to come up with valid solutions by exploring green technology research to tackle this dilemma. In this respect, solar energy has validated itself as an emblematic and efficient approach to addressing energy and water scarcity challenges with minimum environmental impact.^[^ ^6^, ^7^, ^8^, ^9^ ^]^ Efficient solar harvest has been pursued as the oldest and most ubiquitous renewable energy source from the natural evolution of living organisms and is regenerated and updated consistently for more promising applications as an abundant and clean energy resource.^[^ ^10^, ^11^, ^12^ ^]^ Several fruitful attempts are accomplished in designing an effectual solar‐powered freshwater generation to achieve optimized solar‐thermal conversion efficiency to address primitive issues of water shortage.^[^ ^13^, ^14^, ^15^, ^16^, ^17^, ^18^, ^19^ ^]^ However, there still exists a substantial barrier between the status quo and industrial implications owing to the low solar absorption, poor thermal management, and structural deformation due to salt accumulation inside hydrophilic water channels of solar desalination devices when operated under seawater.

Photothermal materials efficiently absorb the incident solar energy over the whole solar‐spectrum range and directly convert it into thermal energy to run the photothermal units.^[^ ^20^, ^21^, ^22^, ^23^ ^]^ Considerably, the heat energy provided by photothermal materials is a concentrated form of energy to boost the charge transportation for photogenerated carriers. The photothermal conversion efficiency is significantly impacted by the solar absorption potential of photothermal material.^[^ ^24^, ^25^, ^26^, ^27^ ^]^ Several photothermal materials have been explored, i.e., carbon‐based materials,^[^ ^17^, ^18^, ^28^, ^29^, ^30^, ^31^, ^32^ ^]^ plasmonic metallic nanoparticle,^[^ ^33^, ^34^, ^35^, ^36^ ^]^ semiconductors,^[^ ^37^, ^38^, ^39^ ^]^ and polymers‐based materials^[^ ^40^, ^41^, ^42^ ^]^ has been reported in the development of efficient solar powered water desalination devices. Lanthanum strontium cobalt iron oxide (La_1−_ *_x_* Sr*_x_* Co_1−_ *_y_* Fe*_y_* O_3_) is a specific ceramic oxide widely used as perovskite material and recognized for its high ionic and electronic conductivities and used as membrane materials for possessing good chemical/phase stability as well as high oxygen permeability.^[^ ^43^, ^44^ ^]^ It has been extensively explored as magnetic,^[^ ^45^ ^]^ catalytic,^[^ ^46^ ^]^ solid oxide fuel cells,^[^ ^47^ ^]^ and dielectric properties. Zhao et al. reported the Lanthanum strontium cobalt iron oxide (LSCF) and 3DOM (3D ordered macro‐porous structure) for synergistic photo/thermos‐catalytic effect for CO_2_ reduction and achieved a high temperature due to the photothermal effect leading toward high efficiency. The high photothermal effect of such materials can also be exploited for enhanced solar steam generation.^[^ ^46^ ^]^ Recently, Ti_3_C_2_ (MXenes), a newly discovered 2D transition metal material has been extensively studied in a variety of applications owing to its excellent electronic conduction with good charge transportation, optimized solar absorption, and numerous hydrophilic groups endorsing its hydrophilicity and strong coupling with other materials.^[^ ^48^, ^49^ ^]^ MXenes are generally represented by the M*_n_*_+1_X*_n_* T*_z_*, where T*_z_* denotes the surface terminations of various groups, i.e., ─O, ─OH, and/or ─F. In fact, these surface terminations facilitate the spontaneous ion intercalation and exchange among 2D nanosheets and bestow it hydrophilicity. More interestingly, these 2D materials can be freely combined with other materials to form layered compounds, which opens the door to the development of novel functional materials and devices.^[^ ^50^, ^51^ ^]^ This approach offers great promise for tailoring the electronic states allowing for more flexibility and tailoring the unique 2D electronic states with atomic‐scale accuracy, allowing for more flexibility in 2D materials and devices by stacking any number of atomically thin layers.

**Figure 1:**
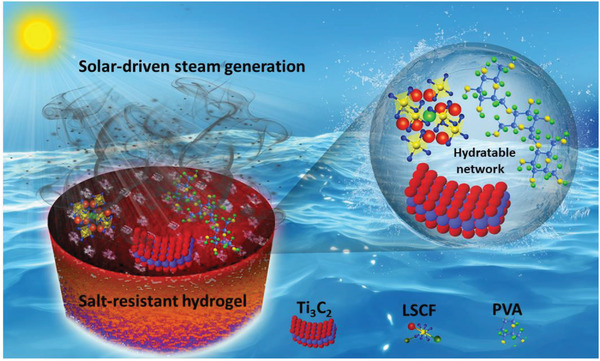
Schematic illustration of 2D MXenes embedded perovskite oxide (La _0.6_ Sr _0.4_ Co _0.2_ Fe _0.8_ O_3−δ_) inspired hydrogels for efficient desalination and stable solar evaporation.

Herein, we report the fabrication of salt‐resistance photothermal hydrogel for seawater desalination prepared by interpenetrating the La _0.6_ Sr _0.4_ Co _0.2_ Fe _0.8_ O_3−_ *_δ_* (LSCF) and Ti_3_C_2_ (MXenes) into a polymeric network of polyvinyl alcohol (PVA) to enable the enhanced photothermal conversion with minimum thermal conduction. The 2D nanosheets of MXene facilitate the maximum light capture with the combination of perovskite oxide nanocomposite. Moreover, skeletons of polymeric networks with the porous channels of hydrogel additives endow the LSCF/Ti_3_C_2_ with ample water content, good thermal management, and high salt resistance for effective solar vapor production, as schematically illustrated in **Figure** **1**.

The prepared device manifested excellent thermal management with a minimum thermal conductivity value of 0.10 049 Wm^−1^ K^−1^ to confine heat over the top surface. The photothermal LSCF/Ti_3_C_2_ hydrogel manifests a high evaporation rate of up to ≈1.98 kg m^−2^ h^−1^ under 1 k Wm^−2^ with ≈90.6% evaporation efficiency. State‐of‐the‐art evaporation performances under different brine concentrations validate its self‐regenerating/rejection efficiency. Based on these merits, the utilization of the perovskite incorporated 2D material composite benefits the enhanced photothermal conversion efficiency to evade surface fouling and achieve long‐term stability (13.33 kg m^−2^ mass change yields during 8 h under one sun) for sustainable seawater desalination.

## 2 Results and Discussion

**Figure 2:**
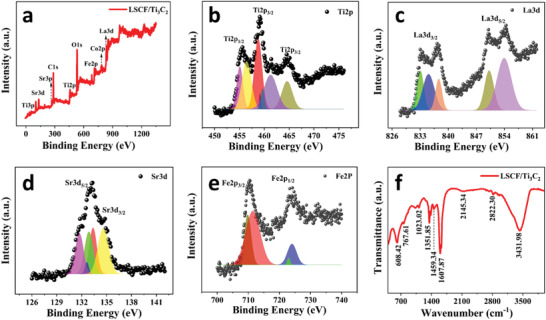
a) Full XPS survey of LSCF/Ti_3_C_2_ powder. b) Highly resolved XPS spectra of b) Ti2p, c) La3d, d) Sr3d, and e) Fe2p. f) FTIR spectrum of LSCF/Ti_3_C_2_.

Harvesting solar energy by photon absorption via a novel combination of materials and collecting the photogenerated hot electrons as a result of internal photoemission has been explored extensively for efficient and stable solar‐driven applications. The LSCF nanoparticles were in situ embedded on the 2D MXenes nanosheets via the facile hydrothermal method. The elemental composition and chemical states of the as‐prepared LSCF/Ti_3_C_2_ powder were affirmed using X‐ray photoelectron spectroscopy (XPS). The whole XPS scan of LSCF/Ti_3_C_2_ is shown in **Figure** **2**a, revealing the presence of the Ti3p, Sr3d, Sr3p, C1s, Ti2p, O1s, Fe2p, Co2p, and La3d, respectively.

The high‐resolution spectrum of Ti2p is shown in Figure 2b. The peak fitting of Ti2p confirms the existence of multiple bonds, i.e., TiO_2_ (459.1 eV), Ti^2+^ (461.21 eV), TiC (464.61 eV), Ti^2+^ (455.47 eV), and TiO_2_ (455.06 eV) due to spin–orbit splitting of 2p into 2p_3/2_ and 2p_1/2_, respectively.^[^ ^19^ ^]^ Figure 2c shows the XPS spectrum of 3d states of La which is split and forms a double structure. Mainly, the spin–orbit coupling causes the La3d states to split into 3d_5/2_ and 3d_3/2_ states. Each state is further split into multiple components 3d_3/2_ (833.09, 835.14, 837.64 eV) and 3d_5/2_ (850.19, 853.96 eV) due to electron transfer to the La 4f from oxygen ligands.^[^ ^19^ ^]^ The XPS spectrum of Sr3d shows multiple components, including 3d_3/2,_ and 3d_5/2,_ components (Figure 2d). The peaks at 131.72 and 132.86 eV correspond to the 3d_5/2_ orbital and at 133.43 and 134.64 eV are attributed to the 3d_5/2_ orbital.^[^ ^19^ ^]^ The 2p peak fitting of Fe was performed according to the constraints for Fe^3+^ and Fe^2+^. The Fe2p spectrum of LSCF shows four prominent peaks at 709.08, 711.03 eV (corresponding to 2p_3/2_ orbital), 722.80, and 723.94 eV (corresponding to 2p_1/2_), as shown in Figure 2e. The existence of the trance quantities of Fe^4+^ could lead the second component of iron (Fe^3+^) to possess relatively higher binding energy values.^[^ ^52^ ^]^ Moreover, the surface functionality of the LSCF/Ti_3_C_2_ was assessed by performing FTIR spectroscopy. The FTIR spectrum of LSCF/Ti_3_C_2_ is given in Figure 2f. The peaks that appeared at 608.42 and 767.61 cm^−1^ are due to the presence of La─O, Sr─O, and H─Sr─OH bonds.^[^ ^52^ ^]^ The peaks from 1000 to 1700 cm^−1^ may be attributed to the O─H bond vibrations and stretching of the C─F bond, respectively. Whereas, the absorption peaks at 2145.34 and 2822.20 cm^−1^ are attributed to the stretching vibrations of asymmetric and symmetric bonds of C─H.^[^ ^53^ ^]^ A wide peak appearing at 3431.98 cm^−1^ is attributed to the stretching band of O─H, indicating the adsorption of water molecules.^[^ ^53^ ^]^

**Figure 3:**
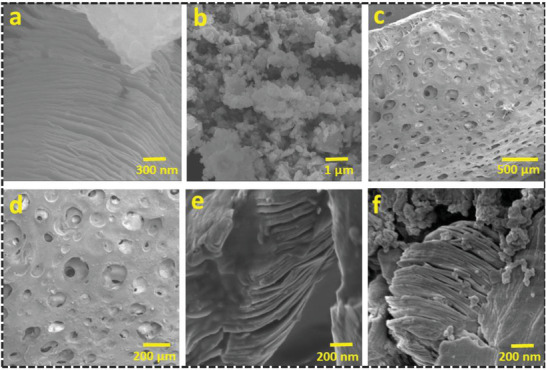
FESEM images of a) 2D nanosheets of MXenes (Ti_3_C_2_), b) perovskite LSCF nanoparticles, and c,d) LSCF/Ti_3_C_2_ hydrogel showing hierarchical pores abundant surface. e) MXene embedded in the polymeric network of PVA hydrogel. f) LSCF/Ti_3_C_2_ nanocomposite embedded in the polymeric network of PVA hydrogel.

The microstructural and surface morphologies of the prepared MXenes (Ti_3_C_2_), LSCF, and LSCF/Ti_3_C_2_ hydrogel were inspected using advanced field emission scanning electron microscopy (FESEM). **Figure** **3**a shows the morphology of the 2D exfoliated MXenes (Ti_3_C_2_) nanosheets which shows the etching using the strong etchant HF aqueous solution to selectively eliminate the aluminum layers, which results in nanoarches at the edges of the exfoliated Ti_3_C_2_ planes. The Van Der Waals forces among 2D planar layers of Ti_3_C_2_ impart a zipping morphological structure that follows a regular and smooth morphology facilitating enhanced surface‐to‐volume ratio and active sites for optimized solar absorption. Figure 3b shows the FESEM image of perovskite LSCF nanoparticles revealing the dense morphology of perovskite material with a rough texture to boost the facile light trapping with intrinsic diffuse reflection and promoted enhanced photothermal effect.

Indeed, the open porous assembly appeals to a better capillary action force for excellent water uptake up to the interfacial surface for vapor generation. The FESEM images of the surface structure of LSCF/Ti_3_C_2_ reveal plenty of hierarchical micrometer‐sized interconnected microchannels to withstand a high potential for quick water supply via capillary force and for vapor generation, as shown in Figure 3c,d. The aligned and ordered symmetry of open pores at both ends of hydrogel ensures efficient water supply, quick vapor escape, and excellent salt rejection. Figure 3e demonstrates the 2D MXene nanosheets embedded in the polymeric network of PVA hydrogel, while the polymeric walls of hydrogels are composed of LSCF/Ti_3_C_2_ nanocomposites which can be seen in Figure 3f. Furthermore, the detailed energy dispersive X‐ray spectroscopy (EDS) also confirmed the presence of LSCF/Ti_3_C_2_ nanocomposites in the polymeric network of PVA hydrogel, as illustrated in Figures S1 and S2 (Supporting Information). The nanocomposite hydrogel depicts the elemental composition which constitutes La, Sr, Co, Fe, Ti, O, N, and C. Additionally, the distribution of LSCF/Ti_3_C_2_ into a polymeric network of PVA entails plasmonic localized heating to augment the solar flux capturing for thermal generation. The hierarchical skeleton of a highly porous assembly can efficiently dissolve the salt ions within the matrix to prevent any accumulation inside aligned microchannels. This anisotropic microchannel also bestows good mechanical strength on the aerogel for structural stability.

**Figure 4:**
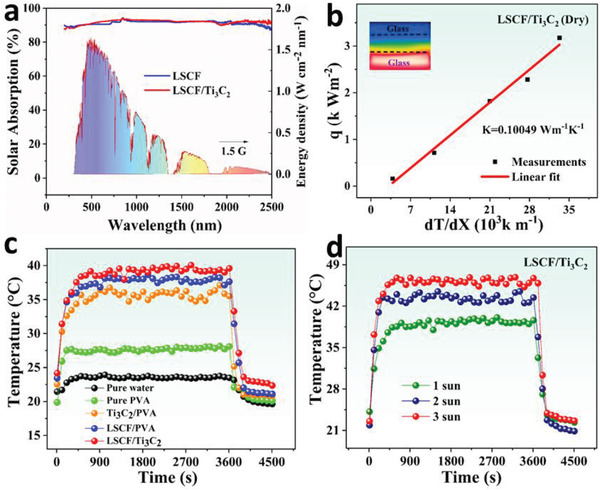
a) UV absorption spectrum of LSCF & LSCF/Ti_3_C_2_ nanocomposite over the entire solar spectral length. b) Thermal conductivity measurement of LSCF/Ti_3_C_2_ hydrogel. c) Surface temperature measurement of the bulk water, Pure PVA, Ti_3_C_2_/PVA, LSCF/PVA, and LSCF/Ti_3_C_2_ hydrogel under 1 sun irradiation. d) Surface temperature evaluation of LSCF/Ti_3_C_2_ hydrogel under 1, 2, and 3 k Wm^−2^ solar intensity.

The efficiency of any designed solar energy‐based system greatly relies on its solar light harvesting potential and its photothermal/photochemical conversion with minimum thermal conduction. We developed an efficient LSCF/Ti_3_C_2_‐based hydrogel that manifests excellent solar absorption over the entire solar spectral length with good solar‐to‐thermal and solar‐to‐chemical energy conversions. The UV–vis spectrum (200–2000 nm) of LSCF and LSCF/Ti_3_C_2_ nanocomposite was carried out using UV–vis spectroscopy (**Figure** **4**a) which shows LSCF/Ti_3_C_2_ nanocomposite exhibits efficient absorption (96%). This efficient absorption potential can be attributed to the localized surface plasmonic resonance (LSPR) of the LSCF/Ti_3_C_2_ which confines the light at the nanoscale and promotes the ultrahigh solar absorption range extended over the entire length of the solar spectrum.

The hierarchical porous structure of the LSCF/Ti_3_C_2_ hydrogel facilitates the diffuse reflections of incident light inside the dark and porous surface to enhance the solar light harvesting potential. Good thermal control can maximize the efficiency of the overall steam generation system. The thermal conductivity of the fabricated LSCF/Ti_3_C_2_ hydrogel was measured experimentally using a thermal conductivity meter (Hot Disk, TPS 2500, Sweden hot disk cooperation). Upon turning on the system, a gradual temperature change (d*T*/d*x*) will develop in a vertical direction creating a temperature gradient. This heat transportation rate (*q*) of the LSCF/Ti_3_C_2_ hydrogel can be use understood using Fourier equations^[^ ^54^ ^]^

(1) $$q\;=\;-k_{1}\;\frac{dT}{dx}=\;-k_{1}\frac{T_{2}-T_{1}}{x_{2}-x_{1}}$$

Where *k*_1_ shows the thermal conductivity (1.05 W m^−1^ K^−1^), *x*_1_ shows the width of the glass slide (3 mm), and *x*_2_ is the LSCF/Ti_3_C_2_ height (30 mm). Where *T*_1_ is the top surface temperature of the thermal conductivity meter, *T*_2_ and *T*_3_ are the bottoms and top temperatures of the glass slides sandwiching the LSCF/Ti_3_C_2_ hydrogel. Finally, the thermal conductivity (*k*) for LSCF/Ti_3_C_2_ hydrogel was computed at the equilibrium point of temperature sustainment and rate by using the following equation

(2) $$k\;=\;q\frac{x_{2}}{T_{3}-T_{2}}$$

The designed LSCF/ Ti_3_C_2_ hydrogel shows minimum thermal conductivity in dry conditions (0.15 069 ± 0.00 792 Wm^−1^ K^−1^) owing to the multiple scattering of incident light to the interior of interfacial surface and transferring their energy in the form of heat to the photothermal surface thereby decreasing the thermal conductivity to the lower matrix of the overall system. The conductivity of the wet LSCF/ Ti_3_C_2_ hydrogel was also measured which come out to be much lower than the thermal conduction of water (0.19 626 ± 0.01 315 W m^−1^ K^−1^) as shown in Figure S3 (Supporting Information).

The interfacial surface temperature enhancement along with minimum thermal conduction plays a decisive role in excellent thermal management. For this, we measured the surface temperatures of the five developed systems, i.e., bulk water, pure PVA, Ti_3_C_2_/PVA, LSCF/PVA, and LSCF/Ti_3_C_2_ to compare the heat accumulation potential by recording the surface temperatures under 1 k Wm^−2^ for 1 h employing two thermocouples positioned on the intended regions, as illustrated in Figure 4c. The LSCF/Ti_3_C_2_ hydrogel manifests the maximum solar harvest, excellent flux distribution within the top matrix, and outstanding thermal management which only allows the water conduction up to the top surface and strictly prohibits the downward thermal conduction. Thus, the interfacial photothermal surface increases rapidly up to ≈40.08 °C and finally reached an equilibrium temperature for the top surface of Ti_3_C_2_/LSCF hydrogel showing a good photothermal conversion rate as the underlying water surrounds the Ti_3_C_2_/LSCF below the interfacial layer, which is up‐taken via porous assembly and then evaporates via liquid–gas phase change. This high surface temperature attainment enables fast steam generation ultimately leading to high efficiency. The surface temperature of LSCF/Ti_3_C_2_ hydrogel was also measured under different simulated solar irradiations and a maximum value of up to 46.27 °C under 3 k Wm^−2^ irradiations (Figure 4d).

**Figure 5:**
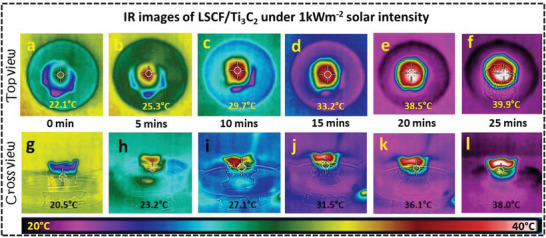
Time‐dependent IR images of LSCF/Ti_3_C_2_ hydrogel during solar evaporation showing thermal distribution for a–f) top surface and g–i) cross surface.

Furthermore, the surface temperature enhancement was also monitored using an infrared (IR) camera for top and cross views under 1 k Wm^−2^ solar intensity. The LSCF/Ti_3_C_2_ (1 cm height and 0.3 cm radius) hydrogel was placed inside a petri dish filled with water under one solar irradiation to capture the temperature for both top and cross surfaces as shown in **Figure** **5**. When the simulated solar intensity was turned on, LSCF/Ti_3_C_2_ hydrogel manifests an immediate photothermal conversion on the top surface by absorbing and converting the incident light into heat energy which ultimately leads to an enhancement in the temperature of the top surface. As shown in Figure 5a–f, the top surface of LSCF/Ti_3_C_2_ hydrogel shows a temperature change of up to 29.7 °C in the first 15 min which is higher than the ambient temperature. In the next 10 min, the upper surface of achieve an equilibrium temperature of 39.9 °C. Whereas, the temperature of the lower matrix of the LSCF/Ti_3_C_2_ hydrogel process maintained a lower temperature value of up to 22 °C throughout this experiment as shown in Figure 5g–l, which was much less than the top surface showing no heat conduction to the downward direction and good thermal insulation for maximum thermal localization on the top surface. The anisotropic low thermal conduction and efficient photothermal conversion of the LSCF/Ti_3_C_2_ hydrogel synergically achieve the optimum “Thermal localization” to achieve a high evaporation rate. Indeed, good solar absorption and photothermal conversion combined with hydrophilicity are contemplated as primary factors to develop a highly efficient steam generation device. The water contact angle test was performed between the water droplet and the surface of the LSCF/Ti_3_C_2_ hydrogel as shown in Figure S4 (Supporting Information), revealing that the LSCF/Ti_3_C_2_ hydrogel can be wetted within ≈0.1 s, demonstrating good hydrophilicity for water transport to achieve high evaporation rate. Moreover, mechanical robustness along with good hydrophilicity is an important factor for the selection of any solar steam generation device for long‐term sustainability. Figure S5 (Supporting Information) represents the physical robustness test of the LSCF/Ti_3_C_2_ which can bear 200 N force without any structural deformation. No surface rupture is observed upon removing the force after 1 h revealing the excellent mechanical stability of our fabricated LSCF/Ti_3_C_2_ hydrogel. All these results indicate that LSCF/Ti_3_C_2_ hydrogel has good application prospects in photothermal evaporation.

**Figure 6:**
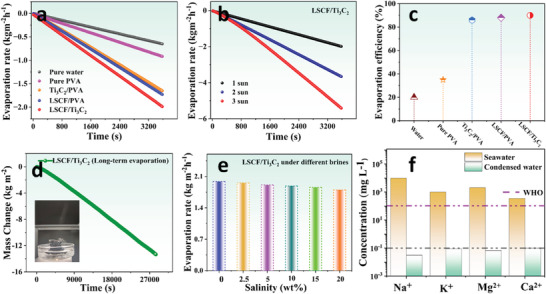
a) Time‐dependent evaporation rate measurement of the developed five systems. b) Evaporation rate evaluation of LSCF/Ti_3_C_2_ hydrogel under multiple solar irradiations. c) Comparative evaporation efficiency analysis of the designed systems. d) Long‐term mass change of the LSCF/Ti_3_C_2_ hydrogel when operated for continuous 8 h under one sun. e) Evaporation rates of LSCF/Ti_3_C_2_ hydrogel under different salt concentration solutions. f) Concentrations of primary salt ions in simulated water and condensed water and condensed water when operated using LSCF/Ti_3_C_2_ hydrogel.

The flux distribution on the top surface of LSCF/Ti_3_C_2_ hydrogel facilitates the excellent photothermal conversion of incident solar light by inherently dispersing it at the interface, which is the basic mechanism of interfacial solar steam generation. Herein, the developed five evaporation systems (i.e., pure water, pure PVA, Ti_3_C_2_/PVA, LSCF/PVA, and LSCF/Ti_3_C_2_) were investigated for the continuous vapor generation under 1 k Wm^−2^ irradiation for 1 h to comparatively analyze their evaporation rate and efficiency. As obvious, the high surface temperature through the LSPR effect with minimum thermal conduction allows enhanced evaporation and elevated evaporation rate. The LSCF/Ti_3_C_2_ hydrogel is recorded for an evaporation rate of 1.98 kg m^−2^ h^−1^ which is the highest among all the other fabricated systems, i.e., pure water (0.65 kg m^−2^ h^−1^), pure PVA (0.91 kg m^−2^ h^−1^), Ti_3_C_2_/PVA (1.64 kg m^−2^ h^−1^), and LSCF/PVA (1.72 kg m^−2^ h^−1^) as shown in **Figure** **6**a. In particular, the interconnected water transportation assembly of microspores provides water up to the top surface quickly and constantly which allows fast vapor escape and efficient thermal distribution on the top surface. At the same time, the inner framework of hydrogel lets in enough light for optimal energy absorption and thermal conversion.

The LSCF/Ti_3_C_2_ hydrogel was also operated under various solar intensities to check its evaporation potential under higher solar irradiations as shown in Figure 6b. Under 3 k Wm^−2^ solar intensity, the LSCF/Ti_3_C_2_ hydrogel shows an evaporation rate of up to 5.41 kg m^−2^ h^−1^ revealing enhanced photothermal response under higher incident light.

The corresponding photothermal conversion efficiency (η_evap_) of the LSCF/Ti_3_C_2_ hydrogel was measured by the following equation^[^ ^50^, ^55^, ^56^, ^57^, ^58^ ^]^

(3) $$\eta_{\mathrm{evap}}=\frac{{\dot{m}}_{v}\;h_{\mathrm{LV}}}{A_{\mathrm{evap}}\int q_{\mathrm{solar}}\left(t\right)d\left(t\right)}\;$$

(4) $$h_{\mathrm{LV}}=\lambda +C\;\Delta T$$

where ${\dot{m}}_{v}\;$ is the net evaporation rate (1.98 kg m^−2^ h^−1^) after substation of evaporation rate under no light condition of bulk water, *h*_LV_ is the liquid‐to‐vapor phase change enthalpy, and *A*_evap_ denotes the evaporation area. Where *λ* here shows latent heat of phase change during the evaporation process (amount of energy required to change the liquid–vapor phase of water during photothermal evaporation) and its value changes at different temperatures (2430 kJ kg^−1^ K^−1^ at 30 °C, and 2256 kJ kg^−1^ K^−1^ at 100 °C), *C* is water‐specific heat capacity (4.2 kJ kg^−1^ K^−1^), △*T* is a temperature difference of water from the initial temperature to the point of vaporization under 1 k Wm^−2^ (surface temperature increment in the course of photothermal evaporation), and *q*_solar_ is simulated solar irradiation, which here is estimated up to 1 k Wm^−2^. The ambient temperature was 23 ± 1 °C and humidity was 55% while performing experiments. Following these equations (iii‐iv), LSCF/Ti_3_C_2_ was calculated for an evaporation efficiency up to corresponding photothermal conversion efficiency (90%) excluding optical and heat losses, which is greater than pure water (20%), pure PVA (35%), Ti_3_C_2_/PVA (86%), and LSCF/PVA (88%) as shown in Figure 6c.

However, the main hindrance being confronted by evaporation structures is the salt accumulation or structural distortion when operated consistently over a long period which significantly influences the evaporation rate and efficiency of devices. For this, LSCF/Ti_3_C_2_ was operated consistently for 8 h under one sun to inspect the smooth evaporation without any structural distortion as shown in Figure 6d. The LSCF/Ti_3_C_2_ hydrogel endows smooth mass change (13.33 kg m^−2^) for 8 h of illumination under one sun. Additionally, to quantitatively assess the evaporation performance, cyclic stability, and durability of LSCF/Ti_3_C_2_ hydrogel, the evaporation rate was also examined under 1 k Wm^−2^ over 10 days, as shown in Figure S6 (Supporting Information). A smooth evaporation rate is maintained by LSCF/Ti_3_C_2_ hydrogel with no significant shift in slope throughout the operating period confirming the excellent stability of the developed system. The corresponding digital images of the LSCF/Ti_3_C_2_ hydrogel are shown in Figure S7 (Supporting Information), reveling no surface degradation after immersing in water for 10 days, exhibiting the good stability of the material and prepared hydrogel in water when operated over a long period. **Table** **1** represents the comparison of solar evaporation performances with other successful evaporators. Furthermore, the LSCF/Ti_3_C_2_ was also operated for salt‐rejection potential with no significant impact on evaporation performance under multiple simulated brine solutions, such as 2.5, 5, 10, 15, and 20 wt%, as shown in Figure 6e. The purifying potential of LSCF/Ti_3_C_2_ in the condensed water was examined against the nanofiltration potential for rejection of primary metal ions using inductively coupled plasma‐optical emission spectrometry (ICP‐OES). Figure 6f demonstrates the primary metal ions, i.e., Na^+^, K^+^, Mg^2+^, and Ca^2+^ concentration in simulated water and condensed. As obvious, the concentration of ions is reduced significantly in the condensed water and surprisingly much lower than present in the standard drinking water set by World Health Organizations (WHOs). Thus, we have successfully developed an LSCF/Ti_3_C_2_ solar evaporator that can be potentially installed for practical applications with no surface degradation and is capable of practical installment at the industrial level for freshwater production.

**Figure 7:**
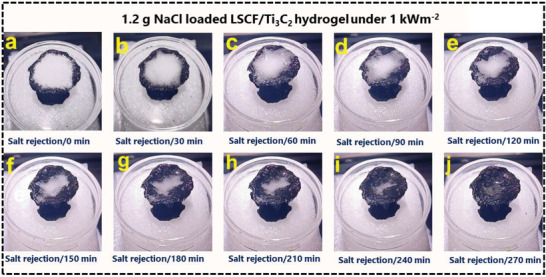
a) Salt‐rejection potential of LSCF/Ti_3_C_2_ hydrogel to analyze the self‐regeneration ability. a−j) 1.2 g of NaCl was spread on the top surface of LSCF/Ti_3_C_2_ hydrogel which was completely rejected back to the bulk water through hierarchical porous assembly within 270 min under 1 k Wm^−2^ solar intensity.

Solar energy, a clean and sustainable power source, is used in the desalination of saltwater. However, because of the high salt content of seawater (3.5 wt% NaCl), the water channels of evaporation structures tend to be blocked during longer operations in colder temperatures. The introduction of novel thin evaporation structures at the expense of thermal insulation is one of several efficient solutions that have recently been suggested to deal with this issue. These thin evaporation structures allow heat to diffuse into the bulk water below from the top interfacial heating layer. The accumulation of salt ultimately prevents the optimization of evaporation efficiency. The self‐regeneration and salt rejection potential of the developed solar‐driven LSCF/Ti_3_C_2_ hydrogel was investigated through different experimental procedures. This was accomplished by placing the LSCF/Ti_3_C_2_ hydrogel in a beaker of salt water (3.5 wt% NaCl) and operating it for several hours at 1 sun solar intensity (1 k Wm^−2^), while dispersing 1.2 g of solid NaCl over the top surface of the LSCF/Ti_3_C_2_ hydrogel, as shown in **Figure** **7**a–j. During 4.5 h of continual irradiation, the LSCF/Ti_3_C_2_ hydrogel rejected all 1.2 g of solid NaCl, demonstrating excellent great self‐regeneration capacity under stable and continuous steam generation. The salt rejection and regeneration capability of the fabricated device is due to the cross‐inked and aligned porous structure throughout the hydrogel. Due to the potential gradient created by the different concentrations in the two places, salt ions will be moved from a high‐concentration to a low‐concentration site by diffusion or convection. The solid NaCl is dissolved by a steady flow of vapor, enabling it to pass through the top surface into the bulk water. The open porous assembly of the LSCF/Ti_3_C_2_ hydrogel is orientated to prevent salt accumulation (Table 1).

**Table 1:** Comparison of solar‐thermal evaporation performance of LSCF/Ti_3_C_2_ hydrogel with other competitive hydrogel‐based solar evaporators

Sr. No	Solar driven system	Evaporation rate [Kg m^−2^ h^−1^]	Efficiency [%]	Absorption [%]	Refs.
1.	SPM‐CH	1.78	90.6	94	[37]
2.	MnO_2_/rGO Aerogel	1.35	93.8	100	[59]
3.	ACET/PAM/SA‐LN(25)	1.64	93.0	95	[60]
4.	Cellulose/alginate/carbon black hydrogel (CACH)	1.33	90.6	97	[61]
5.	LC@LCG	1.84	86.5	98	[62]
6.	Polyelectrolyte photothermal hydrogel	1.69	95.94	95.4	[63]
7.	BiVO_4_‐rGO hydrogel	1.6	87	95	[64]
8.	Biomass hydrogel	1.69	86	98	[65]
9.	CA‐rGO hydrogel	1.47	77	90	[65]
10.	LSCF/Ti_3_C_2_ hydrogel	1.98	90	96	This work

## 3 Conclusion

In summary, we successfully designed nanocomposite hydrogel composed of 2D‐MXenes nanosheets/perovskite La _0.6_ Sr _0.4_ Co _0.2_ Fe _0.8_ O_3−δ_ (LSCF) oxide embedded in PVA matrix for efficient solar‐driven seawater desalination. The LSCF/Ti_3_C_2_ nanocomposite exhibits an aggravated LSPR phenomenon and enhanced surface temperature due to excellent solar absorption (96%). Excellent evaporation rates (1.98 kg m^−2^ h^−1^) under 1 k Wm^−2^ solar irradiation are attributed to the microscale thermal confinement through a hierarchical porous network. Several experiments were performed to evaluate the reproducibility, and stability of the evaporation rate without shape distortion, etc. Using LSCF/Ti_3_C_2_ hydrogel, we were able to realize the salt‐rejection from the top surface (1.2 g NaCl/270 min) without any surface degradation. The present work will provide a techno‐economic analysis to determine if the additional cost of implementing outdoor seawater desalination is worthwhile in our future study.

## 4 Experimental Section

### Materials

All the chemicals lanthanum nitrate (La(NO_3_)_3_.6H_2_O), cobalt nitrate (Co(NO_3_)_2_.6H_2_O), strontium nitrate (Sr(NO_3_)_2_), titanium aluminum carbide (Ti_3_AlC_2_, 98 wt%), and iron nitrate (Fe(NO_3_)_3_.9H_2_O) offered by Wuhan BASF Chemical Industries Co, Ltd., While the citric acid (C_6_H_8_O_7_.H_2_O) and ammonia (NH_3_) took from Wuhan Guangfu fine synthetic industry. Hydrochloric acid ((HCl, GR), chitosan, acetic acid (CH_3_COOH), glutaraldehyde, and absolute ethanol (CH_3_CH_2_OH) were given by Sinopharm Chemical Reagent Co., Ltd. The purchased chemicals were sustaining 99.9% purity, and deionized water was used throughout the whole thing.

### Synthesis of LSCF Nanoparticles

The facile synthesis of LSCF (La _0.6_ Sr _0.4_ Co _0.2_ Fe _0.8_ O_3−_ *_δ_*) was done by following the sol–gel method. Stoichiometric amounts of La(NO_3_)_3_.6H_2_O, Co(NO_3_)_2_.6H_2_O, Sr(NO_3_)_2_, and Fe (NO_3_)_3_·9H_2_O were put in a beaker filled with 300 mL of deionized water to dissolve them. Citric acid C_6_H_8_O_7_·H_2_O was mixed latterly into a nitrate aqueous solution of metal ions as a chelating agent. The ratio of metal ions and citric acid was fixed as 1:1.5. Afterward, to maintain the pH of the solution, NH_3_ was used. Later, the solution was stirred continuously at 80 °C to form a gel and dried at 120 °C for 24 h and followed by sintering the precursor at 1000 °C for 6 h to obtain the LSCF powder. The obtained LSCF powder was ground by putting in a mortar pestle until the formation of fine powder which was saved for characterization and steam generation experimental work.

### Preparation of Ti_3_C_2_ Nanosheets

First of all, 1.0 g of the Ti_3_AlC_2_ was mixed into 100 mL of HF and stirred for 72 h at room temperature. Afterward, vacuum filtration was incorporated to separate the resulting suspension. The acquired product was purified using deionized (DI) water to neutralize the pH. Later it was dried in an oven at 60 °C for 12 h to remove volatile impurities. Then, it was removed from the oven and added to 40 mL ethanol followed by 1 h sonication and 10 min centrifuging at 10 000 rpm. The obtained sediments were again dissolved into ethanol (100 mL), sonicated (750 W, 20 min), and centrifuged (3500 rpm, 3 min) to get desired product which was further dried in an oven at 60 °C to obtain the final powder of ultrathin exfoliated Ti_3_C_2_ nanosheets.

### LSCF/Ti_3_C_2_ Hydrogel

Initially, a stoichiometric amount of PVA (4 g, 10 wt%) was dissolved into the deionized water relatively at a high temperature along with acetic acid to form the homogeneous solution. Then the mixture of LSCF and Ti_3_C_2_ with a ratio of 10:1 was added to the PVA mixture with N_2_ protection. Afterward, 400 µL hydrochloric acid (HCL, 0.1 wt%) was mixed in the prepared solution followed by adding 195 µL glutaraldehyde (4 wt%) and allowed to gelatinize for 6 h. The obtained hydrogel was washed several times in deionized water to get rid of HCl. Afterward, the obtained hydrogel was frozen and defrosted in water (40 °C) two times until it is ready for testing.

### Characterization

The microstructural and morphologies of LSCF powder (La _0.6_ Sr _0.4_ Co _0.2_ Fe _0.8_ O_3−_ *_δ_*), Ti_3_C_2_ (MXene), and LSCF/Ti_3_C_2_ hydrogel were analyzed via Field‐emission scanning electron microscopy (FE‐SEM JSM7100F). X‐ray photoelectron spectroscopy (XPS) was incorporated to affirm the elemental composition of the LSCF/Ti_3_C_2_ composite using THERMO FISHERSICENTIFIC Escalab 250Xi. The light absorption potential of LSCF/Ti_3_C_2_ was checked by performing UV–vis spectroscopy using Shimadze UV–VIS–NIR UV‐3600 double beam spectrophotometer. Further, the existence of multiple functional groups was inspected by conducting Fourier transform infrared (FT‐IR) using an FT‐IR tester (NICOLET iS50, USA).

### Solar‐Driven Evaporation Setup

The solar‐driven steam generation performance of the prepared LSCF/Ti_3_C_2_ hydrogel was checked by operating it under simulated solar intensity using a solar simulator (PLS‐FX300HU) provided with an AM 1.5 solar filter. The intensity of the simulated solar flux was controlled using a CEL‐NP2000‐2 power meter (Beijing Education Au‐light Co., Ltd.). An advanced electronic balance enables measuring with 0.001 g precision (Mettler Toledo, ME204) and was connected to a computer to record the time‐dependent mass variations to measure the evaporation rate. The surface temperatures of different systems of the top layer were recorded using two thermocouples (OMEGA, INSP# 33306, 5TC‐TT‐K‐30‐197) connected with the acquisition system (KEYSIGHT, 34972A). An inductively coupled plasma‐optical emission spectrometry (ICP‐OES, EP Optimal 8000) was employed to measure the concentration of primary salt ions in the simulated and condensed water. All the experiments were performed at room temperature of ≈23 °C and humidity level of *≈*55–50%.

## Conflict of Interest

The authors declare no conflict of interest.

## Supporting information

Supporting Information

## Data Availability

The data that support the findings of this study are available from the corresponding author upon reasonable request.

## References

[1] D. Seckler , R. Barker , U. Amarasinghe , Int. J. Water Resour. Dev. 1999, 15, 29.

[2] A. Boretti , L. Rosa , npj Clean Water 2019, 2, 15.

[3] M. M. Mekonnen , A. Y. Hoekstra , Sci. Adv. 2016, 2, e1500323. 26933676 10.1126/sciadv.1500323 PMC4758739

[4] L. Malaeb , G. M. Ayoub , Desalination 2011, 267, 1.

[5] D. Li , H. Wang , J. Mater. Chem. 2010, 20, 4551.

[6] A. A. Hosseini , S. H. Hosseini , Energy Explor. Exploit. 2012, 30, 389.

[7] V. Devabhaktuni , M. Alam , S. S. S. R. Depuru , R. C. Green II , D. Nims , C. Near , Renewable Sustainable Energy Rev. 2013, 19, 555.

[8] X. Li , W. Xie , J. Zhu , Adv. Sci. 2022, 9, 2104181. 10.1002/advs.202104181 PMC8867196 35018734

[9] C. Ma , W. Wang , Z. Jia , J. Zhang , C. Wang , Energy Technol. 2023, 2300263, 10.1002/ente.202300263.

[10] J. Blanco , S. Malato , P. Fernández‐Ibañez , D. Alarcón , W. Gernjak , M. I. Maldonado , Renewable Sustainable Energy Rev. 2009, 13, 1437.

[11] R. M. Muthusivagami , R. Velraj , R. Sethumadhavan , Renewable Sustainable Energy Rev. 2010, 14, 691.

[12] D. A. Russo , J. A. Z. Zedler , P. E. Jensen , J. Exp. Bot. 2019, 70, 1703. 30773590 10.1093/jxb/erz054 PMC6436153

[13] S. M. Parsa , A. Rahbar , M. H. Koleini , Y. D. Javadi , M. Afrand , S. Rostami , M. Amidpour , Desalination 2020, 491, 114592.

[14] S. M. Parsa , A. Rahbar , D. Javadi Y , M. H. Koleini , M. Afrand , M. Amidpour , J. Clean. Prod. 2020, 261, 121243.

[15] S. M. Parsa , A. Rahbar , M. H. Koleini , S. Aberoumand , M. Afrand , M. Amidpour , Desalination 2020, 480, 114354.

[16] G. Ni , S. H. Zandavi , S. M. Javid , S. V. Boriskina , T. A. Cooper , G. Chen , Energy Environ. Sci. 2018, 11, 1510.

[17] N. Arshad , I. Ahmed , M. S. Irshad , H. R. Li , X. Wang , S. Ahmad , M. Sharaf , M. Firdausi , M. Zaindin , M. Atif , Nanomaterials 2021, 11, 676. 33803494 10.3390/nano11030676 PMC8000500

[18] J. Wang , W. Wang , J. Li , X. Mu , X. Yan , Z. Wang , J. Su , T. Lei , C. Wang , ACS Appl. Mater. Interfaces 2021, 13, 45944. 34525807 10.1021/acsami.1c11176

[19] X. Wang , Z. Lin , J. Gao , Z. Xu , X. Li , N. Xu , J. Li , Y. Song , H. Fu , W. Zhao , S. Wang , B. Zhu , R. Wang , J. Zhu , Nat. Water 2023, 1, 391.

[20] Q. Zhang , X. Xiao , G. Wang , X. Ming , X. Liu , H. Wang , H. Yang , W. Xu , X. Wang , J. Mater. Chem. A 2018, 6, 17212.

[21] Y. Zhang , G. G. Gurzadyan , M. M. Umair , W. Wang , R. Lu , S. Zhang , B. Tang , Chem. Eng. J. 2018, 344, 402.

[22] H. Yin , H. Xie , J. Liu , X. Zou , J. Liu , Desalination 2021, 511, 115116.

[23] T. Ding , G. W. Ho , Joule 2021, 5, 1639.

[24] D. Li , S. Chen , R. Huang , C. Xue , P. Li , Y. Li , Q. Chang , H. Wang , N. Li , S. Jia , S. Hu , J. Yang , Ceram. Int. 2021, 47, 19800.

[25] W. Tao , X. Ji , X. Xu , M. A. Islam , Z. Li , S. Chen , P. E. Saw , H. Zhang , Z. Bharwani , Z. Guo , J. Shi , O. C. Farokhzad , Angew. Chem., Int. Ed. 2017, 56, 11896. 10.1002/anie.201703657 PMC5608550 28640986

[26] N. S. Fuzil , N. H. Othman , N. H. Alias , F. Marpani , M. H. D. Othman , A. F. Ismail , W. J. Lau , K. Li , T. D. Kusworo , I. Ichinose , M. M. A. Shirazi , Desalination 2021, 517, 115259.

[27] T. Gao , X. Wu , Y. Wang , G. Owens , H. Xu , Sol. RRL 2021, 5, 2100053.

[28] C. Wang , J. Wang , Z. Li , K. Xu , T. Lei , W. Wang , J. Mater. Chem. A 2020, 8, 9528.

[29] Y. Lu , T. Dai , C. Lu , C. Cao , W. Zhang , W. Xu , H. Min , X. Yang , Ceram. Int. 2019, 45, 24903.

[30] Y. Xu , Z. Guo , J. Wang , Z. Chen , J. Yin , Z. Zhang , J. Huang , J. Qian , X. Wang , ACS Appl. Mater. Interfaces 2021, 13, 27129. 34098719 10.1021/acsami.1c07091

[31] M. S. Irshad , X. Wang , A. Abbas , F. Yu , J. Li , J. Wang , T. Mei , J. Qian , S. Wu , M. Q. Javed , Carbon 2021, 176, 313.

[32] J. Wang , W. Wang , X. Mu , Z. Li , C. Wang , Sustainable Energy Fuels 2021, 5, 1995.

[33] K. Bae , G. Kang , S. K. Cho , W. Park , K. Kim , W. J. Padilla , Nat. Commun. 2015, 6, 1. 10.1038/ncomms10103 PMC4682046 26657535

[34] M. Gao , P. K. N. Connor , G. W. Ho , Energy Environ. Sci. 2016, 9, 3151.

[35] F. S. Awad , H. D. Kiriarachchi , K. M. AbouZeid , Ü. Özgür , M. S. El‐Shall , ACS Appl. Energy Mater. 2018, 1, 976.

[36] J. Wang , W. Wang , L. Feng , J. Yang , W. Li , J. Shi , T. Lei , C. Wang , Sol. Energy Mater. Sol. Cells 2021, 231, 111329.

[37] M. S. Irshad , X. Wang , M. S. Abbasi , N. Arshad , Z. Chen , Z. Guo , L. Yu , J. Qian , J. You , T. Mei , ACS Sustainable Chem. Eng. 2021, 9, 3887.

[38] M. S. Irshad , X. Wang , N. Arshad , M. Q. Javed , T. Shamim , Z. Guo , H. R. Li , J. Wang , T. Mei , Environ. Sci.: Nano 2022,9, 1685.

[39] M.‐Q. Yang , C. F. Tan , W. Lu , K. Zeng , G. W. Ho , Adv. Funct. Mater. 2020, 30, 2004460.

[40] M. S. Irshad , N. Arshad , X. Wang , H. R. Li , M. Q. Javed , Y. Xu , L. A. Alshahrani , T. Mei , J. Li , Sol. RRL 2021, 5, 2100427.

[41] M. S. Irshad , N. Arshad , J. Zhang , C. Song , N. Mushtaq , M. Alomar , T. Shamim , V.‐D. Dao , H. Wang , X. Wang , H. Zhang , Adv. Energy Sustainable Res. 2023, 4, 2200158.

[42] J. Wang , T. Shamim , N. Arshad , M. S. Irshad , M. N. Mushtaq , C. Zhang , M. Yousaf , L. A. Alshahrani , M. Akbar , Y. Lu , J. Am. Ceram. Soc. 2022, 105, 5325.

[43] Y. Teraoka , Y. Honbe , J. Ishii , H. Furukawa , I. Moriguchi , Solid State Ionics 2002, 152–153, 681.

[44] X. Tan , Y. Liu , K. Li , Ind. Eng. Chem. Res. 2005, 44, 61.

[45] B. Huang , Thermo‐Mechanical Properties of Mixed Ion Electron Conducting Membrane Materials, Forschungszentrum Jülich, Germany 2011.

[46] M. N. Ha , G. Lu , Z. Liu , L. Wang , Z. Zhao , J. Mater. Chem. A 2016, 4, 13155.

[47] H. J. Hwang , J.‐W. Moon , S. Lee , E. A. Lee , J. Power Sources 2005, 145, 243.

[48] L. Chen , X. Mu , Y. Guo , H. Lu , Y. Yang , C. Xiao , Q. Hasi , J. Colloid Interface Sci. 2022, 626, 35. 35780550 10.1016/j.jcis.2022.06.143

[49] Z. Guo , W. Zhou , N. Arshad , Z. Zhang , D. Yan , M. S. Irshad , L. Yu , X. Wang , Carbon 2022, 186, 19.

[50] Z. Lei , X. Sun , S. Zhu , K. Dong , X. Liu , L. Wang , X. Zhang , L. Qu , X. Zhang , Nano‐Micro Lett. 2022, 14, 10. 10.1007/s40820-021-00748-7 PMC8643288 34862938

[51] Q. Zhang , G. Yi , Z. Fu , H. Yu , S. Chen , X. Quan , ACS Nano 2019, 13, 13196. 31633904 10.1021/acsnano.9b06180

[52] A. Rajan , M. Sharma , N. K. Sahu , Sci. Rep. 2020, 10, 15045. 32963264 10.1038/s41598-020-71703-6 PMC7508873

[53] Y. Li , X. Zhou , J. Wang , Q. Deng , M. Li , S. Du , Y.‐H. Han , J. Lee , Q. Huang , RSC Adv. 2017, 7, 24698.

[54] Y. Xu , J. Wang , F. Yu , Z. Guo , H. Cheng , J. Yin , L. Yan , X. Wang , ACS Sustainable Chem. Eng. 2020, 8, 12053.

[55] C. Gao , J. Zhu , Z. Bai , Z. Lin , J. Guo , ACS Appl. Mater. Interfaces 2021, 13, 7200. 33528238 10.1021/acsami.0c20503

[56] Y. Zhu , G. Tian , Y. Liu , H. Li , P. Zhang , L. Zhan , R. Gao , C. Huang , Adv. Sci. 2021, 8, 2101727. 10.1002/advs.202101727 PMC8498870 34382356

[57] L. Song , P. Mu , L. Geng , Q. Wang , J. Li , ACS Sustainable Chem. Eng. 2020, 8, 11845.

[58] Q. Qi , Y. Wang , W. Wang , X. Ding , D. Yu , Sci. Total Environ. 2020, 698, 134136. 31783443 10.1016/j.scitotenv.2019.134136

[59] G. Li , W.‐C. Law , K. C. Chan , Green Chem. 2018, 20, 3689.

[60] J. He , Y. Fan , C. Xiao , F. Liu , H. Sun , Z. Zhu , W. Liang , A. Li , Compos. Sci. Technol. 2021, 204, 108633.

[61] J. Yuan , X. Lei , C. Yi , H. Jiang , F. Liu , G. J. Cheng , Chem. Eng. J. 2022, 430, 132765.

[62] X. Lin , P. Wang , R. Hong , X. Zhu , Y. Liu , X. Pan , X. Qiu , Y. Qin , Adv. Funct. Mater. 2022, 32, 2209262.

[63] B. Peng , Q. Lyu , Y. Gao , M. Li , G. Xie , Z. Xie , H. Zhang , J. Ren , J. Zhu , L. Zhang , P. Wang , ACS Appl. Mater. Interfaces 2022, 14, 16546. 35362947 10.1021/acsami.2c02464

[64] L. Noureen , Z. Xie , M. Hussain , M. Li , Q. Lyu , K. Wang , L. Zhang , J. Zhu , Sol. Energy Mater. Sol. Cells 2021, 222, 110952.

[65] Y. Jiang , N. An , Q. Sun , B. Guo , Z. Wang , W. Zhou , B. Gao , Q. Li , Sci. Total Environ. 2022, 837, 155757. 35525369 10.1016/j.scitotenv.2022.155757

