# Supplementary material for: 2D MXenes Embedded Perovskite Hydrogels for Efficient and Stable Solar Evaporation
Source: Glob Chall. 2023 Jul 20;7(9):2300091. doi: 10.1002/gch2.202300091 (PMC10517291; doi:10.1002/gch2.202300091)
Supplement: Supplementary file 1 — Supporting Information [file GCH2-7-2300091-s001.pdf]

Global Challenges

Open Access

Supporting Information

for *Global Challenges.*, DOI 10.1002/gch2.202300091

2D MXenes Embedded Perovskite Hydrogels for Efficient and Stable Solar Evaporation

Naila Arshad, Muhammad Sultan Irshad, M. Sohail Asghar, Muneerah Alomar, Junyang Tao, M. A. K. Yousaf Shah, Xianbao Wang, Jinming Guo, S. Wageh, Omar A. Al-Hartomy, Abul Kalam, Yabin Hao, Zhengbiao Ouyang* and Han Zhang**

Supporting Information

2D MXenes embedded perovskite hydrogels for efficient and stable solar evaporation

Naila Arshad[a,b], Muhammad Sultan Irshad[a,c], M.Sohail Asghar[c], Muneerah Alomar[d], Junyang Tao[c], M.A.K Yousaf Shah[e], Xianbao Wang[c], Jinming Guo[c], S.Wageh[f], Omar A. Al-Hartomy[g], Abul Kalam[f], Yabin Hao[a,*], Zhengbiao Ouyang[a,*,] and Han Zhang[a,b*]

[a]*Collaborative Innovation Centre for Optoelectronic Science & Technology, International Collaborative Laboratory of 2D Materials for Optoelectronics Science and Technology of Ministry of Education, Institute of Microscale Optoelectronics, Shenzhen University, Shenzhen 518060, P.R. China.*

[b]*Interdisciplinary Center of High Magnetic Field Physics, College of Physics and Optoelectronic Engineering, Shenzhen University, Shenzhen 518060, P.R. China.*

[c]*School of Materials Science and Engineering, Hubei University, Wuhan 430062, P.R. China.*

[d]*Department of Physics, College of Sciences, Princess Nourah bint Abdulrahman University, P. O. Box 84428, Riyadh, 11671 Saudi Arabia.*

[e]*School of Energy and Environment, Southeast University, No. 2 Si Pai Lou, Nanjing 210096, China.*

[f]*Department of Physics, Faculty of Science, King Abdulaziz University, Jeddah 21589, Saudi Arabia.*

[g]*Research Center for Advanced Materials Science (RCAMS), King Khalid University, P.O. Box 9004, Abha, 61413, Saudi Arabia.*

* Corresponding author. Tel.: +86-755-28192679

E-mail: ybhao2020@szu.edu.cn (Yabin Hao)

* Corresponding author. E-mail: zbouyang@szu.edu.cn (Zhengbiao Ouyang)

* Corresponding author. E-mail: hzhang@szu.edu.cn (Han Zhang)

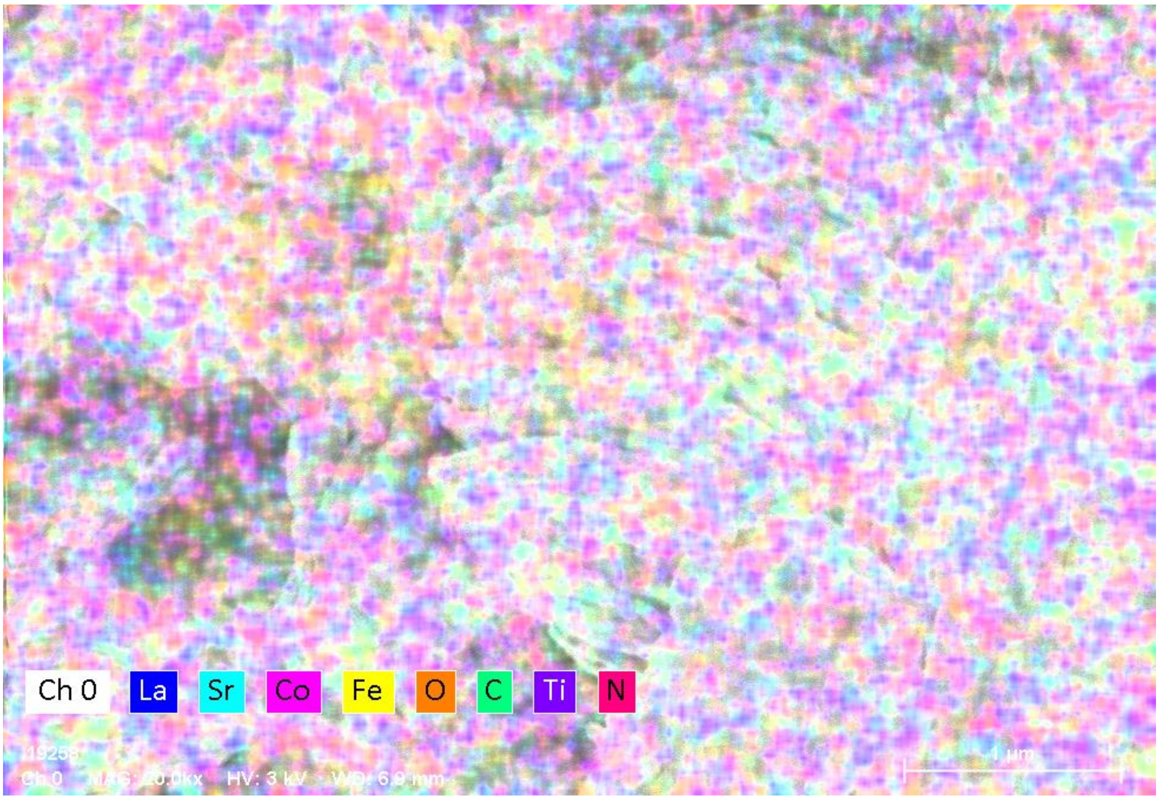

Figure S1. EDS mapping of LSC LSCF/Ti_3C_2 hydrogel.

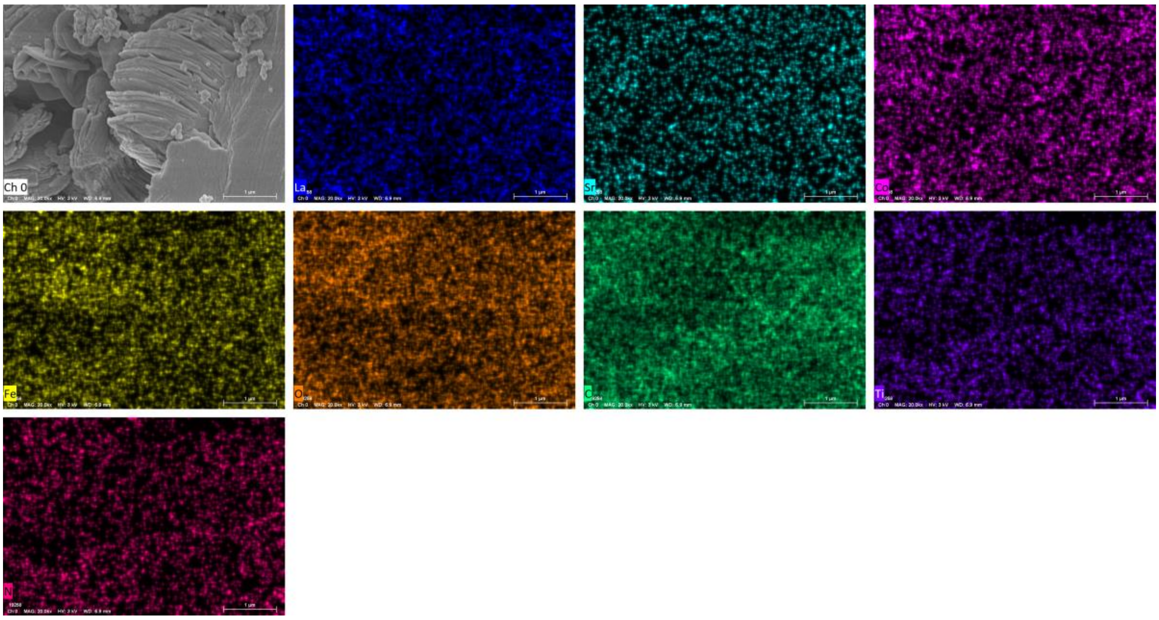

Figure S2. EDS mapping of LSCF/Ti_3C_2 hydrogel which shows the elemental composition such as La, Sr, Co, Fe, Ti, O, N, and C.

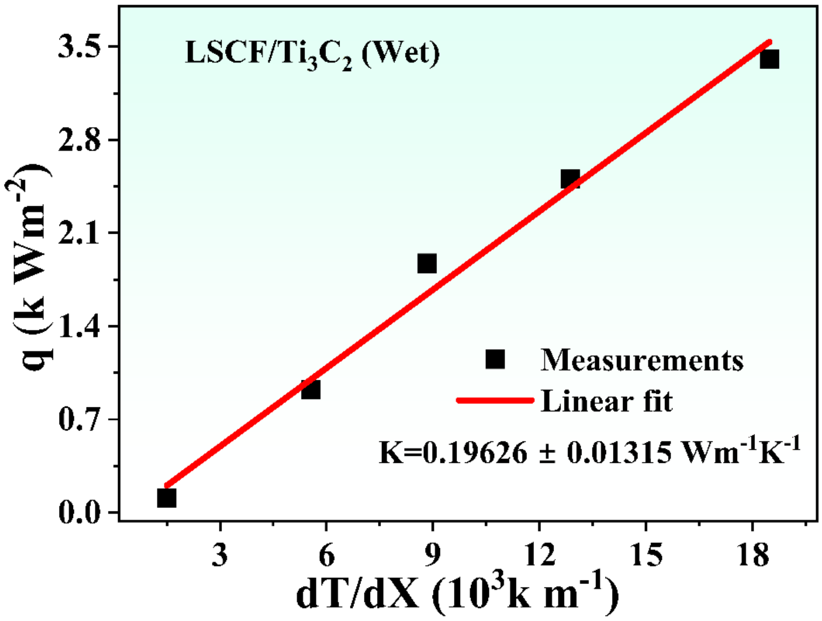

Figure S3. Thermal conductivity of wet LSCF/Ti_3C_2 hydrogel

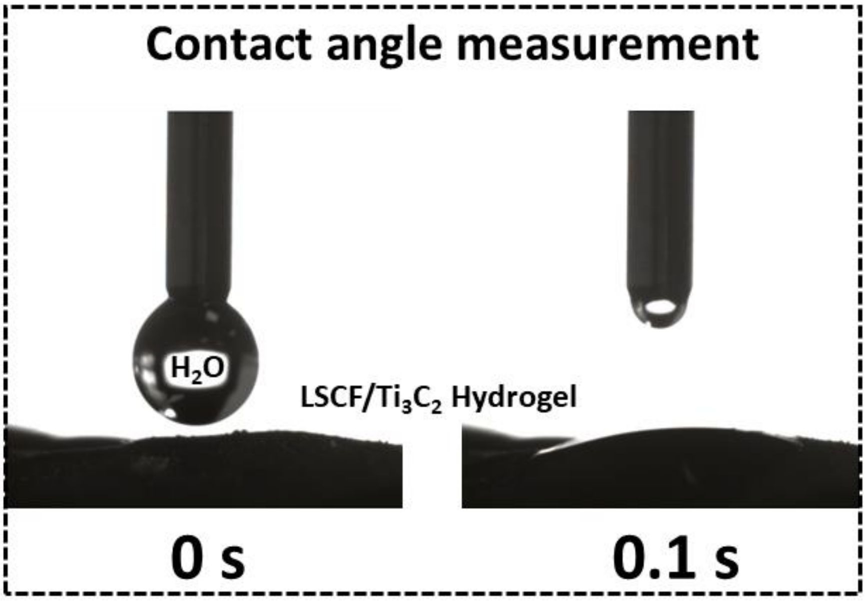

Figure S4. Water contact angle test of LSCF/Ti_3C_2 hydrogel.

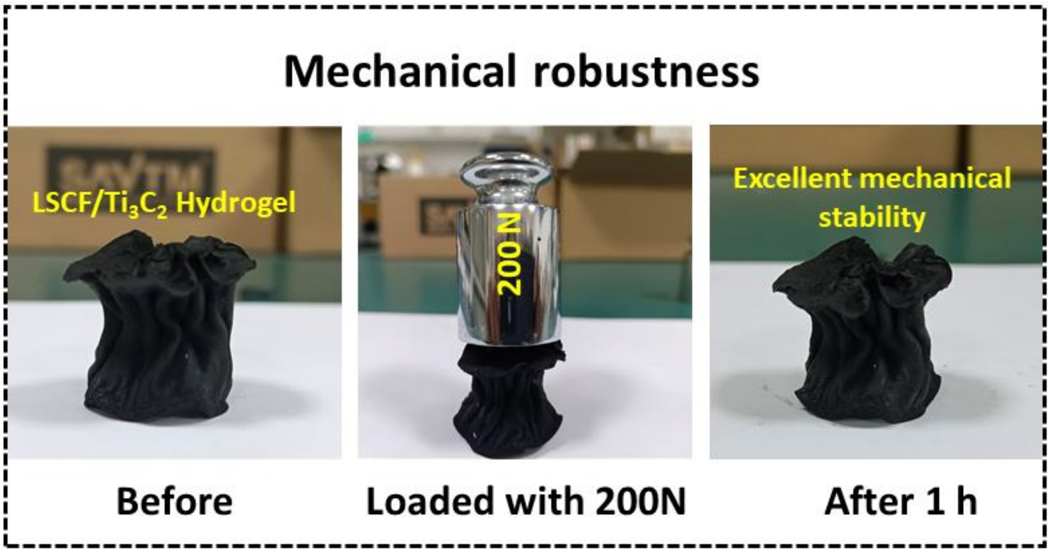

Figure S5. Physical robustness test of LSCF/Ti_3C_2 hydrogel.

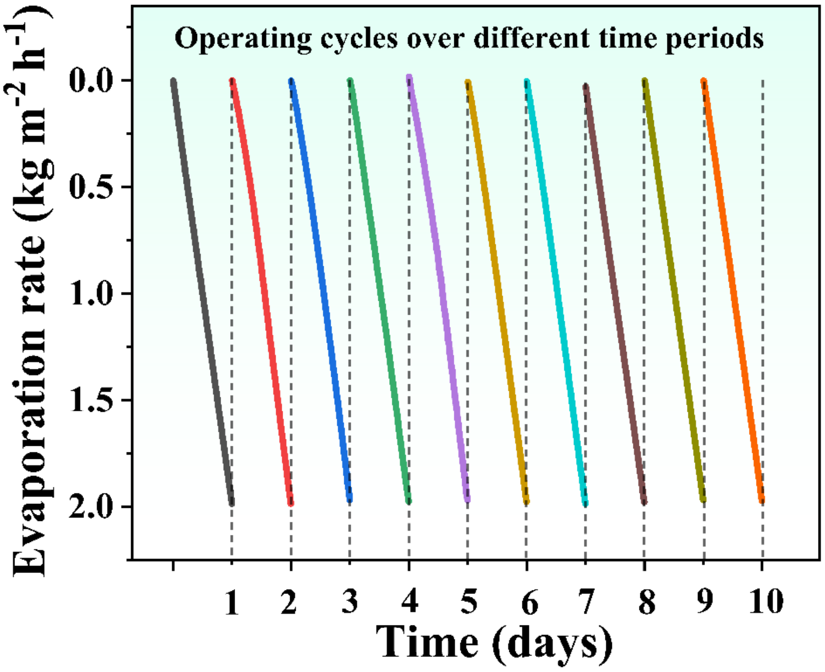

Figure S6. Evaporation rate of the LSCF/Ti_3C_2 hydrogel over different days under one sun.

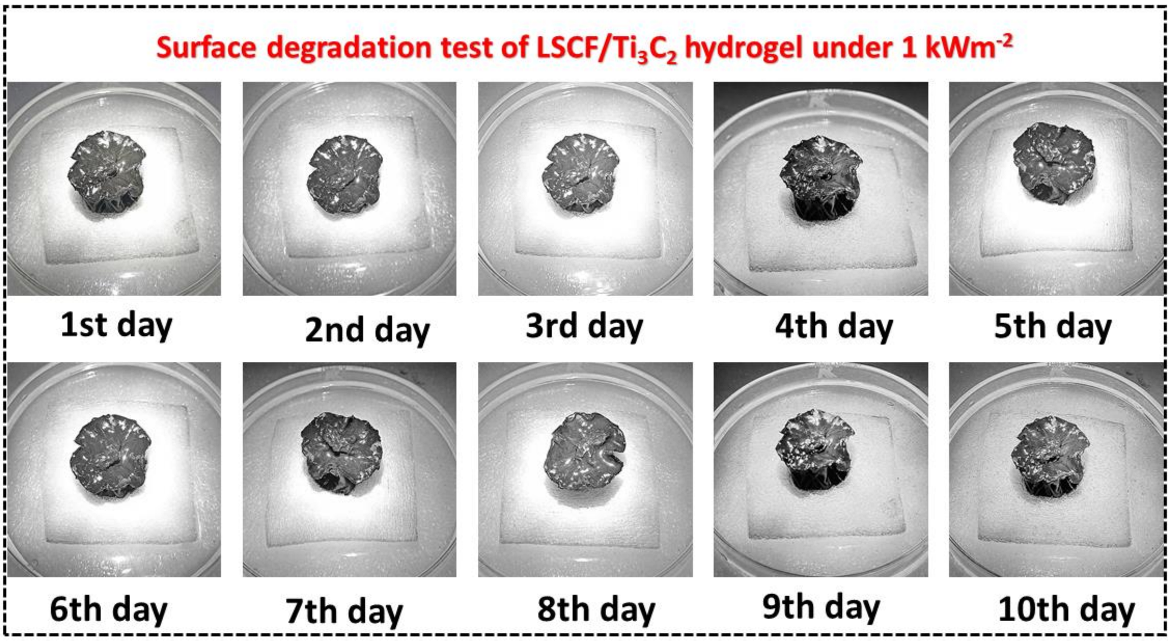

Figure S7. Surface degradation test for the long-term stability of LSCF/Ti_3C_2 hydrogel over different 10 days.
